# Posttraumatic Cavovarus Deformity Due to a Varus Malunited Talar Neck Fracture, Treated With Corrective Talar Osteotomy: A Case Report

**DOI:** 10.7759/cureus.52206

**Published:** 2024-01-13

**Authors:** Ioannis P Galanopoulos, Dimitra Sellina, Panagiotis Drakopoulos, Spyridon A Psarakis

**Affiliations:** 1 Orthopaedics, Thriasio General Hospital of Elefsina, Athens, GRC; 2 Laboratory for the Research of the Musculoskeletal System, KAT Hospital, University of Athens, Athens, GRC

**Keywords:** talar neck fractures, foot deformity, corrective osteotomy, varus malunion, talar malunion

## Abstract

Although talar fractures are frequent bone injuries, fracture displacements of the talar neck are rare, and they can lead to under-treatment and poor prognosis. Furthermore, maltreatment of the talar fractures leads to complications such as malunion (which is the most common), nonunion, osteonecrosis and hindfoot arthritis, which can cause significant disability. The most common position of the talar neck malunion is the varus malunion. Alternative treatments include open reduction with or without bone grafting, open reduction combined with ankle fusion, talar neck osteotomy and talar neck osteotomy combined with subtalar fusion. However, the outcomes of foot function after corrective arthrodesis are poor. In this paper, we present a patient who underwent an open wedge corrective osteotomy of the talus for a cavovarus deformity developed after a malunion of a comminuted talar neck fracture. The patient walked normally three months postoperatively.

## Introduction

Talar fractures are the second most frequent fractures of all tarsal bone injuries [1]. Displaced fractures of the talar neck are uncommon and challenging injuries. Failure to recognize fracture displacement can lead to undertreatment and poor prognosis. In addition, inaccurate reduction of the talar fracture leads to malunion, osteonecrosis and hindfoot arthritis. Malunions or nonunions after displaced talar fractures can cause significant disability because of the hindfoot malalignment in the coronal plane [1]. Malunion of the talar neck is the most common complication of talar neck fractures, and varus malunion is the most common position. The treatment options include open reduction, open reduction with bone grafting, open reduction combined with ankle fusion, talar neck osteotomy and talar neck osteotomy combined with subtalar fusion. Procedures such as corrective arthrodesis do not restore normal foot function.

## Case presentation

**Figure 1:**
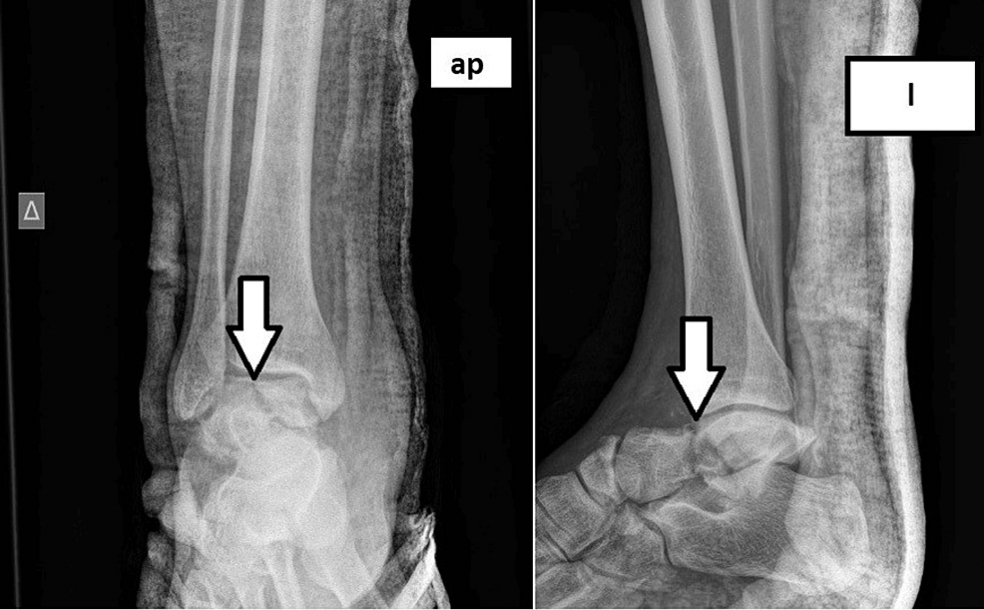
Initial radiographic evaluation depicts a talar neck fracture - Anteroposterior (AP) and Lateral (L) View

**Figure 2:**
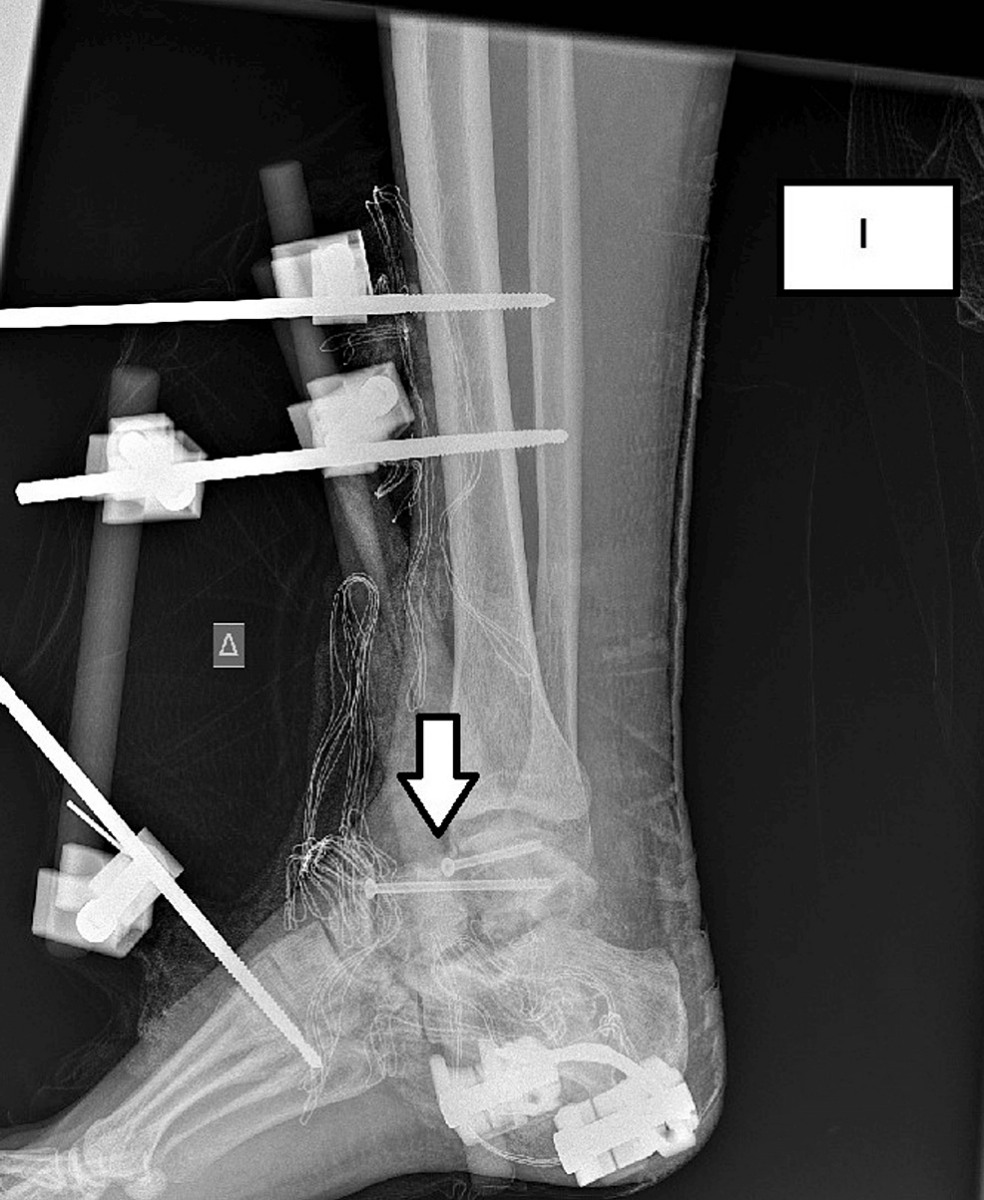
Initial surgery. Internal fixation with two cannulated screws and external fixation Delta frame for additional stability

A 24-year-old patient presented with a comminuted talar fracture-dislocation accompanied by a navicular fracture due to a motor vehicle accident (Figure 1). The patient underwent open reduction and internal fixation with two cannulated 4.5 mm partially threaded screws through the lateral approach of the talus. A Delta frame external fixator was additionally used for extra stability (Figure 2). The patient followed a non-weight-bearing rehabilitation protocol for six weeks. In six weeks, the external fixation system was removed, and the patient started partial weight-bearing in a progressive manner. We have to highlight that in this case, the optimal treatment requires both medial and lateral approaches in order to avoid a varus malreduction.

**Figure 3:**
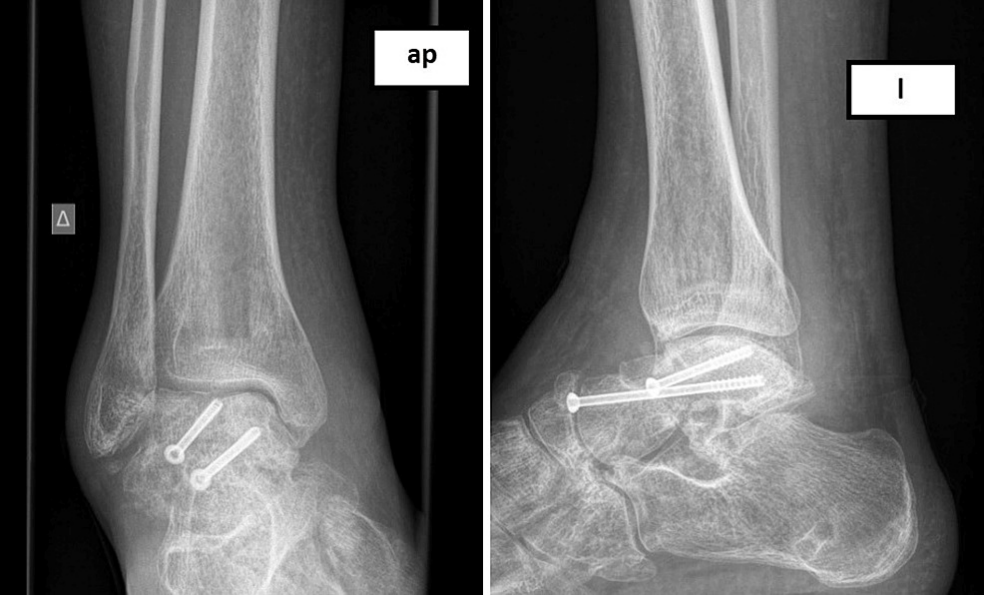
Three months after the initial surgery. The external fixation device has been removed. Anteroposterior (AP) and lateral (L) radiograph showing varus malunion

Three months postoperatively, although the patient walked without crutches, a progressive deformity in a cavovarus position had developed (Figure 3). After a detailed evaluation with computed tomography, a shortening of the medial arch of the foot was recognized. The varus malunion of the talar fracture and the comminution of the navicular bone were responsible for the medial foot column shortening and the following cavovarus deformity of the foot, which led to a lateral foot column overloading during walking.

Six months postoperatively, the talar screws were removed and a new radiographic evaluation was made. No post-traumatic arthritis of the subtalar and tibiotalar joint or talar body necrosis was recognized.

**Figure 4:**
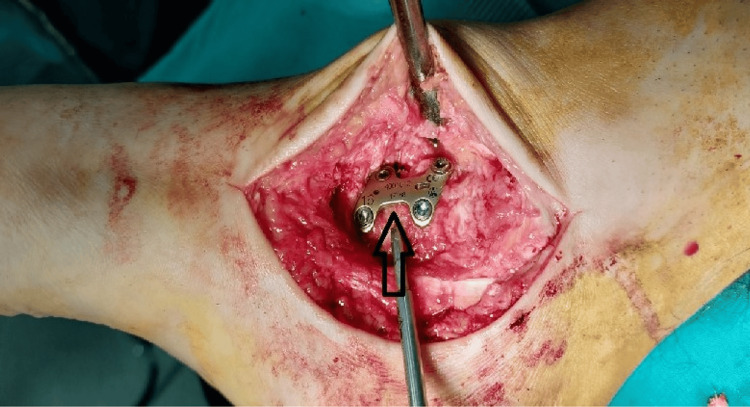
Intraoperative view of the corrective osteotomy. A titanium plate stabilizes the fixation. Tricortical bone graft from the ilium (arrow) restores the length of the talus

**Figure 5:**
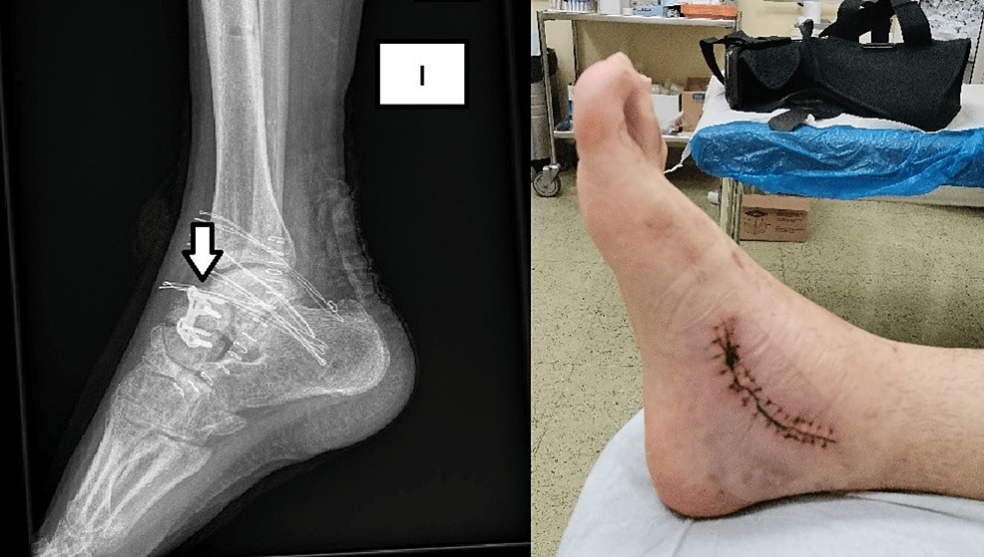
Final radiological picture (left side) and clinical picture of the foot (right side) 20 days after the corrective osteotomy of the talus. No cavovarus deformity exists.

Nine months postoperatively, the patient underwent a talar corrective osteotomy. The aim of the surgery was to restore the length of the medial column of the foot and the correction of varus malalignment of the talus. Under general anesthesia, in a supine position, a medial approach to the talus was performed. A wide chisel was used for the osteotomy at the side of the malunion. After a complete osteotomy, a laminar spreader was used to correct the shape of the talus, forming a wedge in which an autologous tricortical bone graft from the ilium was inserted. Following that, a specific, titanium, 4-hole plate was placed to secure the construction (Figure 4). Postoperatively, a non-weight-bearing protocol was followed and the leg was placed in a splint for eight weeks. After eight weeks, the splint was removed, and the patient started progressive, partial weight-bearing. Twelve weeks postoperatively, the fracture had fully healed, and the patient started walking normally and returned progressively to his daily activities (Figure 5).

## Discussion

In this case report, a 24-year-old patient with cavovarus deformity and medial foot column shortening due to a varus malunion of a talar fracture underwent a talar corrective osteotomy restoring the medial column length using tricortical bone graft and a titanium plate. Twelve weeks postoperatively he returned to his daily routine. The main goal of this case report is to highlight that the initial treatment requires both medial and lateral approaches in order to avoid a varus malreduction. Furthermore, in the case of varus malunion and progressive cavovarus deformity, a corrective osteotomy can be a viable option. There are variable options in the literature about this problem.

Rammelt and Pitakveerakul [2] address the crucial concern of avoiding posttraumatic varus deformity. This study highlighted the importance of early and accurate diagnosis, along with appropriate management strategies, to prevent or minimize the development of varus deformity following hindfoot injuries.

An essential diagnostic technique for assessing the alignment of the hindfoot is the Hindfoot Alignment View, which was described by Saltzman and el-Khoury [3]. This radiographic view can diagnose and monitor conditions affecting the hindfoot, such as fractures, deformities, and arthritis. However, this complication still remains not rare.

Sanders et al. [4] investigated functional outcomes after displaced talar neck fractures. They attempted to comprehend the functional implications of these fractures, providing valuable insights into the potential challenges and outcomes associated with displaced talar neck fractures.

Huang and Cheng [5] discussed the management of neglected or malreduced talar fractures, emphasizing delayed surgical treatment as a viable approach. The authors analyze the challenges associated with addressing these fractures after initial neglect or improper treatment, highlighting the principles of anatomy and biomechanics of the talus. They reviewed various surgical techniques, such as open reduction in three cases, open reduction with bone grafting in three cases, open reduction combined with ankle fusion in one case, talar neck osteotomy in one case and talar neck osteotomy combined with subtalar fusion in one case, tailored to the specific characteristics of neglected or malreduced talar fractures, focusing on the careful preoperative planning and the patients’ profile. The approach was medial to the tibialis anterior, initiating at the navicular tuberosity. This study contributes valuable insights into the complexities of managing talar fractures that have been neglected or inadequately treated, providing guidance for orthopedic surgeons facing such challenging conditions.

Rammelt et al. [6] conducted a prospective study including 10 patients, focusing on anatomical reconstruction of malunited talar fractures. Through diligent observation and intervention, the researchers examined the long-term outcomes, providing valuable insights into the success and durability of anatomical reconstruction techniques for talar malunions. Some patients were treated with open reduction with either K-wires or screw fixation, while others received nonoperative treatment. In some cases, corrective osteotomy was performed, and autologous bone grafting was used. Additional interventions encompassed the release of adhesions in the ankle and subtalar joints, revising the sinus tarsi, and performing extensor tenolysis. The findings contribute to the evolving understanding of effective strategies for managing malunited talus fractures and may inform future clinical practices in this demanding orthopedic domain. In addition, professionals [7] explored the risks involved in corrective procedures performed after the initial treatment of talar fractures, emphasizing the complexities and potential pitfalls in such interventions.

Lamm et al. [8] contributed research to examine the use of distraction osteogenesis, specifically the Gigli saw midfoot osteotomy technique combined with external fixation, in addressing complex foot deformities. Both podiatrists and orthopedic surgeons collaborated and focused on innovative surgical approaches. The authors aim to contribute to the understanding and advancement of treatment options for intricate foot deformities through the application of Gigli saw midfoot osteotomy and external fixation procedures.

Monroe and Manoli [9] presented a case report illustrating the successful application of osteotomy as a treatment approach for the malunion of a talar neck fracture. Through an anteromedial approach, the talar neck was exposed, and osteotomy with an oscillating saw and osteotome was performed. A laminar spreader determined the optimal position, confirmed by radiograph after correcting the hindfoot varus. A rhomboid-shaped autogenous tricortical iliac crest bone graft was placed into the osteotomy. Internal fixation with a cortical screw, retrograde from the medial talonavicular joint corner, secured the graft, avoiding compression to prevent osteotomy shortening. The study underlines the effectiveness of this procedure in correcting malunion, emphasizing its potential as an applicable option for managing talar neck fractures with associated deformities.

Suter et al. [10] described a surgical technique involving a talar neck osteotomy by lengthening the medial column to manage malunited fractures in the talar neck. A dorsomedial approach was performed, and implant removal preceded correctional osteotomy in some cases. Posteromedial releases addressed tendon contractions and additional Achilles tendon lengthening was needed in few patients. Correctional measures involved the use of a Hintermann TM distractor, and grafts, primarily human cancellous allograft blocks, were inserted. Stability was achieved with screws or buttress plates, considering factors such as bone graft adequacy. The technique requires precision in osteotomy placement to restore proper anatomical alignment, ultimately enhancing foot function and mitigating issues associated with malunion. The collaboration of these professionals highlights the importance of surgical intervention for complex talar neck fractures.

Zwipp and Rammelt [11] focused on secondary reconstruction procedures for malunions and nonunions of the talar body. A bilateral strategy is often necessary, with an anteromedial approach for the medial and central aspects. A separate anterolateral approach is used for the subtalar joint and lateral process. Surgical steps include assessing cartilage status, excising nonviable fragments, performing osteotomies, and using screws or plates for fixation. Techniques such as curettage, drilling, and bone grafting are employed as needed. Overall, the study provides insights into the complexities of talar body malunions and nonunions and suggests alternatives for effective secondary reconstruction procedures, potentially contributing valuable information to the field of orthopedics.

## Conclusions

The corrective, open wedge osteotomy with additional tricortical bone graft and internal fixation with plate and screws seems to be a reliable technique to restore the malalignment and the secondary foot deformity after a varus malunited talar fracture. This option is not viable in case of tibiotalar or subtalar joint arthritis and talar necrosis.

## References

[1] Bykov Y (2014). Fractures of the talus. Clin Podiatr Med Surg.

[2] Rammelt S, Pitakveerakul A (2019). Hindfoot injuries: how to avoid posttraumatic varus deformity?. Foot Ankle Clin.

[3] Saltzman CL, El-Khoury GY (1995). The hindfoot alignment view. Foot Ankle Int.

[4] Sanders DW, Busam M, Hattwick E, Edwards JR, McAndrew MP, Johnson KD (2004). Functional outcomes following displaced talar neck fractures. J Orthop Trauma.

[5] Huang PJ, Cheng YM (2005). Delayed surgical treatment for neglected or mal-reduced talar fractures. Int Orthop.

[6] Rammelt S, Winkler J, Heineck J, Zwipp H (2005). Anatomical reconstruction of malunited talus fractures: a prospective study of 10 patients followed for 4 years. Acta Orthop.

[7] Rammelt S (2012). Secondary correction of talar fractures: asking for trouble?. Foot Ankle Int.

[8] Lamm BM, Gourdine-Shaw MC, Thabet AM, Jindal G, Herzenberg JE, Burghardt RD (2014). Distraction osteogenesis for complex foot deformities: Gigli saw midfoot osteotomy with external fixation. J Foot Ankle Surg.

[9] Monroe MT, Manoli A II (1999). Osteotomy for malunion of a talar neck fracture: a case report. Foot Ankle Int.

[10] Suter T, Barg A, Knupp M, Henninger H, Hintermann B (2013). Surgical technique: talar neck osteotomy to lengthen the medial column after a malunited talar neck fracture. Clin Orthop Relat Res.

[11] Zwipp H, Rammelt S (2016). Secondary reconstruction for malunions and nonunions of the talar body. Foot Ankle Clin.

